# Company Representatives’ Experiences of Occupational Health Surveillance for Workers Exposed to Hand-Intensive Work: A Qualitative Study

**DOI:** 10.3390/ijerph18042018

**Published:** 2021-02-19

**Authors:** Kristina Eliasson, Gunilla Dahlgren, Therese Hellman, Charlotte Lewis, Peter Palm, Magnus Svartengren, Teresia Nyman

**Affiliations:** 1 Department of Medical Sciences, Occupational and Environmental Medicine, Uppsala University, Uppsala University Hospital, 751 85 Uppsala, Sweden; therese.hellman@medsci.uu.se (T.H.); peter.palm@medsci.uu.se (P.P.); magnus.svartengren@medsci.uu.se (M.S.); teresia.nyman@medsci.uu.se (T.N.); 2 Department of Public Health and Clinical Medicine, Section of Sustainable Health, Umeå University, 901 87 Umeå, Sweden; gunilla.dahlgren@umu.se (G.D.); charlotte.lewis@umu.se (C.L.)

**Keywords:** ergonomics, legislation, musculoskeletal disorders, medical health checks, qualitative research, risk assessment, work environment

## Abstract

Exposure assessment seldom precedes the medical health checks in occupational health surveillance. In order to emphasize the interconnection between exposure assessment and medical health checks, a process model was developed. The process model aimed to guide employers and Occupational Health Service providers through the execution of occupational health surveillance. The objective of this qualitative study is to explore company representatives’ experiences of the process model, in terms of feasibility and values, and to identify factors that facilitate or impede the process. Thirty-three company representatives from ten companies were interviewed. Interviews were analyzed using content analysis. The company representatives experienced that the model contributed to increased risk awareness and understanding of the exposure effects on workers’ health. They valued the exposure assessments performed by an ergonomics expert, which led to the discovery of previously unidentified risks. The feasibility was facilitated by: a joint start-up meeting in which the process was planned, clear communication between the involved parties, and clarity regarding the process ownership. The findings reveal that a guiding process model is valuable for the execution of occupational health surveillance. However, the model should not only define the components included; a practical guide concerning how the process can be executed is also needed.

## 1. Introduction

The ultimate aim of occupational health and safety regulations and legislation is to prevent and reduce the number of fatalities, injuries, and disorders caused by work hazards, as well as to promote a sustainable healthy work environment [1,2]. Andersen et al. (2019) conclude in a review that both general and specific legislation are effective incentives to improve the work environment and to reduce fatalities and injuries [3]. Specific legislation includes regulations about occupational health surveillance, which encompass medical health checks tailored to specific hazardous exposures [4,5].

The most common perception of occupational health surveillance is that they foremost serve the purpose of protecting the individual worker from ill-health. There are several approaches for achieving this goal: (1) removal of hazardous exposures, (2) early detection of ill-health, and (3) restricting exposure for sensitive individuals (removing the individual from the work task). However, occupational health surveillance also serves as an indicator of hazardous work environments, and thereby they are an important part of occupational health and safety management (OHSM) in an organization.

Employers have the responsibility to ensure that the workplace meets the requirements stipulated in the National Work Environment Act [1,2]. To aid with the occupational health surveillance, the employer can engage an external work environment expertise such as an Occupational Health Service (OHS) provider. However, evaluation of legislated occupational health surveillance for different exposures has shown that exposure assessment seldom precedes the medical health checks of exposed workers and that companies rarely involve their OHS provider in the exposure assessment [6,7,8]. This procedure means that OHS-provider might lack information about the exposure that the medical health check should target. This results in that measures of occupational health surveillance only target the individual and that work environmental measures are overlooked. Reports also indicate that occupational health surveillance seldom results in preventive actions/work environment improvements [8]. There may be several explanations for this, among others that there is a lack of practical guidelines related to the execution of work environmental provisions and policy documents [9], and that occupational health and safety issues are not integrated into the organization’s management systems but are treated as a “side-car” [10,11,12].

### Musculoskeletal Disorders and Occupational Health Surveillance

Musculoskeletal disorders are a major cause for sick leave and work disability in Sweden as well as in Europe [13,14,15,16]. Nevertheless, occupational health surveillance has traditionally targeted chemical or physical workplace hazards. However, several musculoskeletal disorders in the neck and upper extremities are associated with manual work, high hand forces, and repetitive hand-intensive work [17,18,19,20,21,22,23,24]. Carpal tunnel syndrome (CTS), for example, is a diagnosis that is strongly associated with hand-intensive work, a common diagnosis causing sick leave [16]. Recently, guidelines on threshold limit values for exposure–response relationships for occupational physical exposures regarding musculoskeletal disorders in the neck and upper extremities have been suggested [25,26]. This may contribute support to the employer concerning the risk assessment.

A few studies have evaluated comprehensive ergonomics programs targeting musculoskeletal disorders (such as CTS) related to hand-intensive exposure. May (2002) evaluated the implementation of an “OHSA Local Emphasis Program” in New Hampshire aiming at the reduction of ergonomics hazards relating to CTS. The results indicated that the companies in the program reduced their incidence of CTS during a period of several years after the intervention [27]. Another study described the “Washington State ergonomic rule”, which focused on reducing workplace hazards that could cause, or aggravate, musculoskeletal disorders. All employers with “Caution Zone Jobs” (awkward postures, high hand forces, highly repetitive motion, repeated impact, heavy frequent or awkward lifting, or hand/arm vibration) were covered by the rule. The evaluation showed that the rule resulted in reduced exposures, with positive effects on injury incidence and absenteeism; however, following the rule’s repeal, the exposures increased [28]. Seemingly, occupational health and safety legislation and regulatory systems can be important incentives to encourage employers to take action for preventive interventions [3,29].

The association between exposure to hand-intensive work and musculoskeletal disorders in the neck and upper extremities has been acknowledged by the Swedish Work Environment Authority who recently introduced specific occupational health surveillance targeting hand-intensive work [30]. This is unique legislation in that it is the first Swedish occupational health surveillance targeting a specific ergonomic exposure.

**Figure 1:**
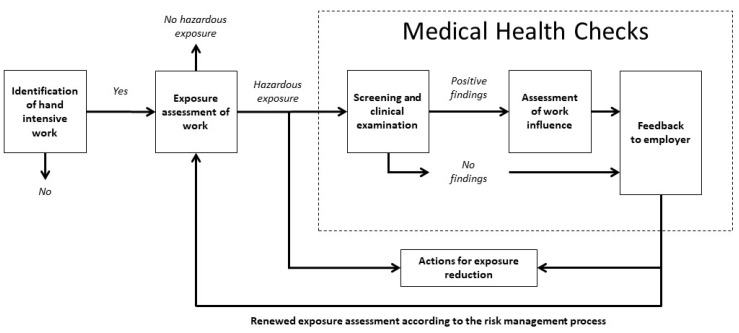
The work process model for the execution of occupational health surveillance for workers exposed to hand-intensive work (HIW)-model. The model interconnects exposure assessments with medical health checks.

Figure 1 illustrates a newly developed model that describes the work process execution for occupational health surveillance of workers exposed to hand-intensive work, the (HIW-model). The development of the process model as well as the description of the different components of the model is presented by Eliasson et al. [31]. The process model aims to guide the employer (as having the legal responsibility for the work environment) and the OHS provider (an independent expert supporting employers regarding the work environment and health-related issues) through the work process of occupational health surveillance. The HIW-model is designed to follow the structural outline of a risk management process: Identification, Assessment, Control, and Monitoring of exposure [32,33] and encompasses the following main components; identification of hand intensive work and exposure assessment followed by medical health checks of exposed workers. By interconnecting the findings from the exposure assessment with the findings from the medical health checks to an overall assessment of if and how the identified exposures are associated with findings of musculoskeletal disorders among exposed workers, a comprehensive risk assessment can be made. This comprehensive risk assessment (based on information both regarding exposure and outcome) forms the basis for exposure reducing actions, which in turn should be controlled and monitored according to the periodical risk management process.

Information about occupational health surveillance programs targeting musculoskeletal risk factors is scant, although international studies point to the need to implement prevention programs to reduce the prevalence of upper-limb musculoskeletal disorders MSDs [27,28,34]. However, there is a knowledge gap regarding the practical work (the execution) of occupational health surveillance regarding the work process as well as the collaboration between the employer and the expert provider of risk assessment and medical health checks in such process. Employers are responsible for the implementation and execution of occupational health surveillance. Knowledge regarding their experiences on the execution of occupational health surveillance by following a structured work process, like presented in the HIW-model, is not earlier explored. Such knowledge is important at an overall level to achieve information regarding the factors that are important to consider for effective implementation of an occupational health surveillance program. Given the new occupational health surveillance regulation regarding hand-intensive work introduced by the Swedish Work Environment Authority, it is of interest to explore the feasibility and values of the HIW-model. Results from such an exploration may render important knowledge to support the development of implementation guidelines.

The aim of this study is to explore company representatives’ experiences of occupational health surveillance for hand-intensive work when following the HIW-model. The focus is on how the company representatives experience the in-work process according to the HIW-model, in terms of feasibility and values, and to identify factors that facilitate or impede the execution of the work process.

## 2. Methods

This study is part of a larger research project, which explores the stakeholders’ experiences of the HIW-model and if and how the process model has any impact on actions for the reduction of exposure to hand-intensive work in the workplace [31]. In the study presented in this paper, we used a qualitative exploratory design. The data collection was based on semi-structured interviews. The Consolidated criteria for Reporting Qualitative research (COREQ) checklist [35] was used to support the reporting of this study.

The Regional Ethical Review Board in Uppsala approved the study (project reference number 2017/274).

### 2.1. Company Selection

Participating companies were selected through purposive sampling [36] of companies with workers exposed to hand-intensive work. The intention was to include a heterogeneous group (both geographical and size) of companies from different sectors. Several companies were contacted via telephone or e-mail, and provided with information about the study. In total, 30 companies were invited to participate in the study. Ten companies accepted participation in the study, thirteen companies rejected participation, and seven companies did not reply. The most common reason for rejection was lack of time.

The ten companies were located in the northern (four), middle (three), and southern (three) regions of Sweden. The following sectors were represented: Assembly (4) (e.g., truck components, technology products, Automation); painting (1); cleaning (1); food handling (1); Dental Technology (1); Foundry (1) (manual material handling, grinding); and Dairy (1) (goods handling). The companies were small, (*n* = 2, <50 employees), medium (*n* = 4, >50), and large (*n* = 4, >250). Concerning the large companies, the whole company did not take part in the project; the participating units were smaller units within the company. However, the partaking units shared the OHSM system with the whole company.

Each company independently formed a project group, consisting of company representatives (e.g., first-line manager, Health, Safety and Environment (HSE) manager, safety representative). Each of the groups was supplemented with the ergonomist from the company’s OHS provider (in-house *n* = 1, external *n* = 9). The ergonomists had professional background as registered physiotherapist (*n* = 9) or naprapath (*n* = 1). An overview of the companies and their project groups is presented in Table 1.

### 2.2. Study Informants

The criteria for inclusion was that the informant was a member of the project group in one of the included companies. Each company could decide for themselves which and how many representatives they wanted to involve in the project; however, they were informed that at least one manager with responsibility for the work environment and one safety representative should be participating in the project group. Each participant was informed about the aim of the study and was given both written and verbal information about their participation. All of them signed an informed consent form. In total, 36 company representatives participated in the present study, and data pertaining to the representatives are presented in Table 1. Twenty-six (16 men, 10 women) with a mean age of 43 (range 27–64) had a role at the managerial level. Ten (8 men, 2 women) were safety representatives, with a mean age of 45 (range 34–56).

### 2.3. Study Context

The participating companies were instructed to execute the HIW-model process according to their own prerequisites. The process started with joint start-up meetings, one in each region, with the companies’ project groups. A meeting included a presentation and a “walk-through” of the HIW-model, as described in Eliasson et al. (2020) [31]. The research project had pre-set requirements concerning who should execute some of the components in the model (Figure 1). It was set that the company representatives should execute the identification of hand-intensive work tasks at the workplace without the involvement of the ergonomist. The exposure assessment of the identified hand-intensive work tasks and the medical health checks were to be executed by the ergonomist, whose services were economically reimbursed according to the existing financial agreement between the company and the OHS provider.

### 2.4. Data Collection

The process was explored through individual interviews and focus group interviews with the companies’ representatives (Table 1) on two occasions during a 6-month period. The interviews followed semi-structured interview guides. K.E. and G.D. (both Ph.D. students, registered physiotherapists, and ergonomists with professional experience of OHS) constructed the interview guides. The respective guide drafts were discussed several times in the research group until final versions were agreed upon. The final versions were then piloted tested and slightly modified thereafter.

The first interview focused on exploring the execution of the different components; from identification to medical health checks. For example, a question could be “Please, tell us about how you executed the identification of hand-intensive work”, followed by a probing question that explored more details how the specific component was executed. Next, the question was about how the representatives experienced the components exposure assessment, etc., and the collaboration with the ergonomist.

The second interview was more comprehensive, and explored the company representatives´ experiences of the HIW-model as a whole. The interview also included questions regarding the execution of the different components, as well as questions about the process, e.g., collaboration with the ergonomist and facilitators and barriers for execution.

Representatives from all ten companies participated in the interviews. However, not all of the 36 (Table 1) representatives participated in all interviews because of various reasons. Thirty-three (92%) representatives participated in at least one interview, and twenty-three (64%) participated in both interviews. The first interview was held as an individual telephone interview with each company representative. In total, 28 representatives participated, approximately 2.5 months after the start-up meeting. At that point, most of the companies had recently completed the exposure assessment and medical health checks; however, only a few had received a feedback report from the ergonomist at that time. The duration of the telephone interviews ranged from 15–45 min and they were conducted individually either by K.E. or by G.D. The second interview was a face-to-face focus group interview, conducted at the premises of each company, approximately 6 months after the completion of the HIW-model. In total, 28 representatives participated in the second interview. The size of the focus groups varied from two to five informants. In one company, only one person could participate in the face-to-face interview; thus, the other participant was interviewed by phone individually. In total, 11 interviews were conducted at the second data collection point. The focus group interviews ranged from 30–90 min and were held by an interviewer-pair, with one interview moderator and one observer that asked supplementary questions. Either K.E. or G.D. was always one of the interviewer-pairs; they conducted interviews together or were accompanied by either T.N., C.L., or P.P. In total, 39 interviews were conducted, and all interviews were digitally recorded and transcribed verbatim.

### 2.5. Data Analysis

The interviews were analyzed by qualitative content analysis with a manifest focus inspired by Graneheim and Lundman (2004) [37] and Elo and Kyngäs (2008) [38]. The transcribed interviews were coded using QSR International’sNVivo 12 software. The interviews were analyzed separately for each company (*n* = 10), and brought together into one text. The analysis process started with the first author thoroughly reading through one focus group interview. Thereafter, the coding started by marking condensed meaning units of the text. The condensed meanings units were phrased close to the informants’ own wording. The condensed meaning units were labelled into different sub-categories. A deductive approach [38,39] was used to gather condensed meaning units related to the different components in the model. Each component in the model constituted overarching sub-categories. Condensed meaning units related to the execution process were categorized based on an open inductive approach [38,39,40]. The condensed meaning units were compiled into a text for the focus group interview. Thereafter, the interviews from the first data collection point related to the company were read through, new findings were coded, and the text was extended. Most often, these interviews did not add any new information; however, they added trustworthiness [37,41] to the data by validating the information from the second data collection point. Interviews from two to three companies were compiled into one text, thereafter the authors K.E. and T.N. met and discussed and compared the findings and sub-categories, and if necessary, re-visited the interviews relating to each company. The entire process was repeated for all ten companies. Finally, K.E. compiled the ten texts into one draft of preliminary findings that was peer-reviewed by T.H., P.P., and M.S. This group was inter-professional and represented a variety of different experiences and perspectives. To have this group peer-review the findings further strengthened the reliability of the analysis and minimized the risk of the analysis being characterized by the pre-understanding of K.E. and T.N. The findings were discussed with K.E. and T.N. in order to reach a consensus [42]. Thereafter, the results were presented for G.D. and C.L., who had conducted some of the interviews, and they confirmed that the findings supported their experience of what was being expressed in the interviews.

## 3. Results

The findings are presented in two categories. The first category describes the company representatives’ experience of the HIW-model components; identification, exposure assessment, screening and clinical examination and feedback. The second category describes their experiences of facilitating factors and barriers for the execution of the HIW-model. An overview of the categories and sub-categories is shown in Table 2.

### 3.1. Model Components

#### 3.1.1. Identification of Hand-Intensive Work

The objective of the identification was that it should be simple and straightforward for an employer to execute. The sole purpose of this component was to ascertain whether the workers were (or were not) exposed to hand-intensive work. In fact, the identification should only render a yes/no answer to the question: “Are the workers, in their current work environmental conditions, exposed to hand-intensive work or not?” [31].

Even though it was obvious that workers were exposed to hand-intensive work in all of the participating companies, (this was an inclusion criterion to be included in the project), the interviews revealed that several informants, especially from companies with a greater variety of work tasks, expressed uncertainty regarding the identification of hand-intensive work and whether the work tasks could be considered hand-intensive enough. They expressed a lack of guidance and support on how to assess and evaluate factors such as exposure time, work-rotation, and repetitiveness of work tasks. Informants also expressed a desire to involve the ergonomist already in this phase, which indicated both an uncertainty among the representatives concerning their own competence, and a wish for this component to be more detailed. The informants highlighted the importance of the identification component, pointing out that the findings define when, and for what work tasks, a comprehensive exposure assessment is needed. Some informants underlined the importance of a clear definition of what should be regarded as high enough exposure for hand-intensive work to be subject to the following exposure assessment component according to the model. They suggested that examples from different work tasks and from various sectors could help to guide the employer in the identification of hand-intensive work.

#### 3.1.2. Exposure Assessment of Work

The objective of the exposure assessment was to assess, analyze, and quantify hazardous exposures to provide the information needed for deciding which workers were to be subjected to the medical health check (screening and clinical examination). The exposure assessment should be performed by the ergonomist [31].

##### The Expert Validity

The informants were all familiar with the concept of general workplace risk assessments, as being part of the company’s OHSM system. However, the practice of commissioning an external expert for a targeted exposure assessment assignment varied between the companies. Several informants perceived the exposure assessment as reliable and comprehensive when it was conducted by a commissioned expert with competence within the field of ergonomics. Even in those cases when the informants already seemed to be aware of most of the hazardous work tasks that were analyzed in the exposure assessment, they still expressed that they valued the expert’s assessment since it added credibility when confirming the informants’ own perceptions of hazardous work tasks. As one informant explained:

“It feels good to have the view from an outsider. You easily get blind to flaws at home. One does not really see the risks as an outsider does, so I think it has been valuable.”

(Representative from company 9)

Further, several informants highlighted the importance of the exposure assessment as the results may demarcate the basis for risk reduction actions at the workplace.

##### Increased Risk Awareness and Workplace Learning

The informants expressed that the exposure assessment led to increased risk awareness among both workers and managers since previously unidentified ergonomics hazards were identified and exposure levels determined. One informant from a company that handled goods of low weights but that had highly repetitive work tasks explained that:

“We never thought that we could get work related disorders in the same way as those with heavier work. But even though we work with lighter products, we have a high risk, and that has not been so obvious until now. And that is really good!”

(Representative from company 8)

Another informant reported that through the ergonomist’s comprehensive exposure assessment, the company became aware of additional concerns, such as stress-related issues. The first line manager described that the workers “opened up” to the ergonomist and shared concerns with the ergonomist that they had not raised earlier even though the company regularly and systematically worked with work environmental issues. The manager in question, who considered himself to have a good and close relationship with the employees, initially became a little annoyed. However, in time, he appreciated that these previously unknown hazardous factors were raised so that they could plan for actions to be taken.

Several informants conveyed that the ergonomists involved both workers and company representatives in performing the exposure assessment. Some informants expressed that such a participatory approach increased knowledge at the workplace regarding ergonomic exposure assessments and insights into ergonomic assessment tools. As one informant explained:

“Some of these models were new to us; it was the first time so to speak. So, it becomes like an educational part also.”

(Representative from company 5)

#### 3.1.3. Screening and Clinical Examination

The objective of the screening and clinical examination was to examine the neck and upper extremities, with the aim of identifying pain and disabilities among those workers who, according to the exposure assessment, were exposed to hazardous levels of hand-intensive work [31].

Most often, the examinations were performed at the premises of the company, which the informants experienced as both time-efficient and convenient. Informants also highlighted that this approach enabled worker participation since they did not have to leave the workplace to partake in the examination. The informants described that information of the results from the clinical examination increased their knowledge that hand-intensive work tasks can cause musculoskeletal disorders not only in the hand, but also in the entire upper extremities as well as in the neck. One informant pointed out that their project group found the clinical examination enlightening, since they all had had the misapprehension before that hand-intensive work only affects the hands.

“The concept hand-intensive is a bit misleading, I think, if you look at the clinical examinations and the discomforts, you do not have immediate pain in your hands.”

(Representative from company 5)

The informants described that they already, before the clinical examinations, had relatively good insights concerning the health of their employees. Some informants discussed whether it might be just as informative to investigate the workers’ work-related musculoskeletal disorders with a survey, instead of subjecting the employees to a clinical examination. However, in the end, they found the clinical examination added valuable aspects, such as how work can have an impact on health. The results from the examinations made them aware of early signs of musculoskeletal disorders among their workers, which prompted them to take action immediately. These actions could either target the individual worker (e.g., further examinations or treatments) or target the work organization (e.g., re-design of production flow). The informants reasoned that such insights could not be reached through a survey. However, some considered it a possibility to replace the clinical examination with a survey every other time the occupational health surveillance for hand intensive work was repeated.

Another valuable aspect, which was stressed by the informants, was that they felt it was valuable to offer the clinical examinations to the workers. According to the informants, the workers appreciated, during the examination, getting individual advice from the ergonomist concerning their disorder, physical activity, and work technic training.

“…You find that you have problems there; it is almost too late anyway, but at least it is so that more people will manage in the future... You might find if it is work-related and if so what we should do to avoid it tomorrow...”

(Representative from company 7)

#### 3.1.4. Feedback

The objective of the feedback was that the ergonomist should inform the employer whether the previous exposure assessed work tasks could be related to the identified musculoskeletal disorders in the exposed workers. The feedback should also propose actions to reduce exposures and suggest actions for workers with musculoskeletal disorders [31].

None of the informants had any previous experience of medical health checks that targeted specific exposures in the work environment. They described the feedback phase as encouraging them to pay attention to their employees, both concerning their health and the work environment. One informant explained that the feedback highlighted the individuals and not merely the hazards, which could facilitate the implementation of risk-reducing actions. The informant described that when awareness is focused on the individuals performing the work task, rather than on the work task itself, it gives a better understanding of the interconnection between work exposure and its effects on workers, as it is easier to identify with the worker than with only an exposure measurement.

##### Structure and Presentation of Feedback

The feedback to the company representatives was in most cases provided as a written report followed by an oral presentation, an arrangement that the informants appreciated. Several informants described that they valued a detailed and tangible report in which the focus was on the results and proposed actions and not on how the exposure assessment and clinical examination were executed. They described that the feedback should include the current exposure levels, prevalence of work-related musculoskeletal disorders among the workers, and action proposals to reduce the exposure and prevent musculoskeletal disorders. To include details about what assessment tools the ergonomist had used in the exposure assessment was perceived as less important. Some informants underlined that when the feedback contains proposals for actions, it is important that the ergonomist also explains what the expected outcomes of the action proposals might be.

“You do not need to know what all of these assessment tools are called and what numbers they have resulted in. But what is it that they have seen and what are we supposed to do. A little more straight forward!”

(Representative from company 3)

##### Feedback—Valuable for Dissemination

The informants stated that the feedback phase was important for the dissemination of information and that the feedback contributed to increased awareness among both workers and union representatives. Therefore, some expressed the importance of the feedback (both written and oral) being adapted to the audience (e.g., workers, union representatives, health and safety committees), in order to simplify distribution within the company or corporate group. Some informants had chosen to invite the ergonomist to present the feedback to the workers. The informants expressed that the feedback to the workers could contribute to increased understanding and acceptance among the workers, since their awareness was raised about how hazardous work tasks could affect their own and their co-worker’s health. In some companies, the feedback led to employees starting to encourage each other to be more aware of the correct work technique.

### 3.2. Facilitating Factors and Barriers for the Execution of the Model

#### 3.2.1. Planning and Preparation

The joint start-up meeting was highly appreciated, as each project group (the company representatives with their respective ergonomists) was given time to plan their individual process. For several of the companies, it was a new experience to prepare and plan together with the company’s ergonomist.

At the end of the start-up meeting, all project groups had set a rough timeframe for the execution of the exposure assessment and the clinical examination. The follow-up interviews revealed that for most of the companies, the timeframe was held. However, time for the feedback was not set in advance which, for some, resulted in the feedback being delayed and given much later, or even not at all. Informants expressed that in this type of project it is important to have a coherent process with a distinct timeframe and pre-determined deadlines.

#### 3.2.2. Communication and Roles

Overall, the process ran without difficulties despite not having explicitly defined project roles and ways for communication in advance. However, in companies where these elements were unclear, the process was hampered. This was especially evident in one company where the informants were unsatisfied with their process, expressing that the process dragged on and that they did not hear anything from the ergonomist regarding the progress. In that company, the workers were located at different geographical sites, and the company representatives explained that the ergonomist had difficulties reaching the workers. The company had not allocated the role of coordinator to a specific person. The person that could be considered to have the role of process owner was located at the corporate group’s national headquarters, far from the local site where the model was executed. In hindsight, acknowledging their shortcomings regarding coordination and communication, they expressed that they should have organized their process in another way.

“I think [it had been helpful] that steering it up a bit more in the beginning and also, you might have had some regular meetings as well.”

(Representative from company 2)

Another example of deficiencies in communication was found in two companies, in particular. The informants described that they had not informed the workers about the objective and content of the clinical examination. It came as a surprise for the workers that they were to undergo a clinical examination. In one company, the workers disapproved of the examination in the beginning. However, when the ergonomist and the manager explained the intention and the procedure of the examination, the workers accepted participation and in the end, all of them were very satisfied.

“...in the communication, the language… there was a little to begin with, but when that was resolved, then there was like no weirdness but it worked well after that. So, that was exactly it and it is probably these small cultural differences we have that you have to work a little with.”

(Representative from company 9)

In the follow-up interviews, informants highlighted the importance of arranging information meetings with the involved project members and workers continuously during the process.

“Invest in information before, during, after, get a group that becomes responsible for this, and that it will result in something so that you are willing to take action based on what the results show.”

(Representative from company 10)

One company highlighted a way to assure the spread of information. They had organized their process such that after each phase, they arranged a short feedback meeting in which the project group (the ergonomist and the company representatives) met and verified what was done and what would happen next. This procedure, recurrently updating everyone in the project group, was appreciated and made it possible to disperse information to the workers about the progress of the process. After the completion of the HIW-model and after the project group had taken part in the feedback report, the project owner dispersed information about the project and its outcomes to different management groups within the overarching company group. The informant experienced that this approach contributed to anchor the action proposals and to establish a structure for future medical health checks for hand-intensive work in their company.

#### 3.2.3. Collaboration

The informants expressed that the collaboration with the ergonomist was important and facilitated the process. They emphasized factors such as: the ergonomist’s experience, the relationship between the company and the ergonomist, and the ergonomist’s specific knowledge about their company or sector. Another important factor was the flexibility of the ergonomist, so that the company could customize the time schedule for the exposure assessments and clinical examinations to suit the production. On the other hand, one informant expressed that earlier collaboration with the ergonomist was not a necessity, implying that the importance was to have an ergonomist that is experienced and possesses sector knowledge.

#### 3.2.4. Outer and Inner Contextual Factors

During the interviews, it emerged that both inner and outer contextual factors influenced the process. In the outer context, all informants emphasized that legislation will be a fundamental factor for the execution of the HIW-model, and the informants were hesitant to execute a similar process without legislative pressure. Furthermore, some informants reflected that being part of a research project facilitated the process as it implied that the company was supportive and that the project was prioritized.

In the inner context, the informants believed that having committed senior managers and interested first-line managers in the company also facilitated the process.

“It has also obviously made it easier to have a head of department who actually wants to do this, because otherwise it is very difficult to carry out this in different departments.”

(Representative from company 10)

Furthermore, some of the informants highlighted that it was also supportive when workers were positive and engaged in the project. Another inner contextual factor was the maturity and structure of the company’s OHSM system. Several of the companies worked actively with work environmental issues, and they already had a structure, where this new medical health check could be incorporated. Inner structural facilitating factors mentioned by the informants were, for example, already being accustomed to the regular risk and workplace assessments, having a prioritized focus on safety at work, and having a manifest health and safety organization.

## 4. Discussion

In this qualitative study, we explored company representatives’ experiences of a process model for the execution of occupational health surveillance, targeting hand-intensive work (HIW-model). Information from company representatives´ regarding feasibility and values as well as usability aspects of the HIW-model is important in order to acquire knowledge of their needs for how the work with occupational health surveillances should be organized and executed.

The focus in the HIW-model is the interconnection between the exposure assessment and the medical health check (Figure 1) [31]. The company representatives appreciated the guidance provided through the model. The HIW-model contributed to increased risk awareness and understanding of how individual musculoskeletal disorders were related to the work environmental exposure. The representatives valued the exposure assessments being performed by an external expert, with competence in ergonomics because unidentified risks were ascertained and aggravating exposures such as stress were elucidated. The feedback component was important for the dissemination of the exposure assessment within the organizations and in some cases resulted in spontaneous attempts among workers to reduce the risk. The execution of the HIW- model was facilitated by: a joint start-up meeting in which the process was planned, clarity regarding the ownership of the process, and supportive management. However, the fundamental incentive for the execution was the legislation.

An unexpected finding was the company representatives’ uncertainty regarding the hazard identification. This indicates that more support and guidance is needed for company representatives to execute this step with confidence, e.g., to use screening tools. A screening tool should have high sensitivity, meaning that the risk of misclassification is inclined toward an overestimation of potential risk. There are existing screening tools that can be used by company representatives without specific ergonomic training. One example of such a tool is the Washington State Ergonomic checklist [43]. When supervisors and workers have used this tool, it has been shown to be sensitive enough to identify risks; however, the risk has been overestimated compared to assessments performed by a trained ergonomist [43]. Since the identification of hand-intensive work is followed by a more in-depth exposure assessment in the HIW-model, the proper risk level would be estimated in that step. Thus, in the present study, the company representatives may have been guided by using such a screening tool.

**Figure 2:**
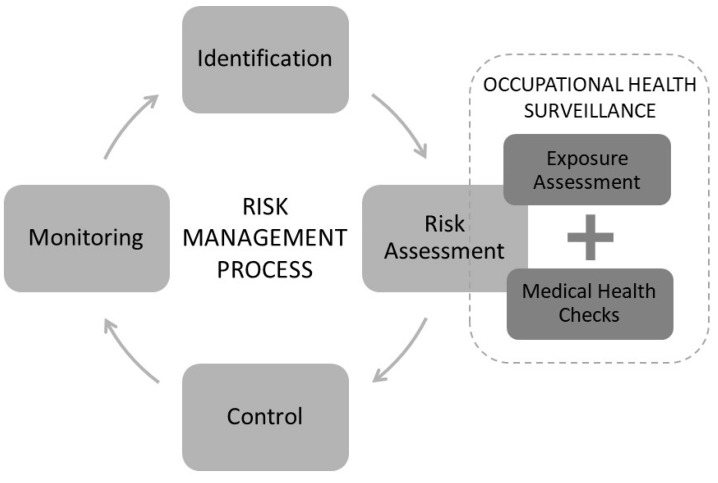
Exposure assessment and medical health checks targeting the current exposure constitute a comprehensive risk assessment in the risk management process.

Occupational medical health checks should be based on an assessment of a specific exposure [4,5,30]. However, reports indicate that this is not always the case [8]. In this study, we focus on the interconnection between exposure and health outcome (musculoskeletal disorders). This interconnection was a new experience for the informants. This finding points out the importance of clarifying the aim with the occupational health surveillances for employers, so that the exposure assessment is the basis for a targeted medical health check, where those together provide information that constitutes an immersed risk assessment about how the work environment impacts the workers’ health. Figure 2 below visualizes the place of the occupational health surveillance as a part of the ongoing risk management process.

The representatives illuminated that the interconnection between exposure assessment and medical health checks resulted in a clear and realistic picture of how the work affects musculoskeletal disorders. It was emphasized that the awareness of musculoskeletal disorders was an important factor for the company to plan for preventive actions in the work environment. This finding is supported by Martinson et al. (2014), who reported that knowledge of workers’ health, as well as the capability to evaluate relevant outcome measures after a workplace intervention (which is possible if an exposure assessment is made both prior to and after risk controlling actions), were incentives to take actions at the workplace [29]. A similar result is presented by Yazdani (2018), who suggests the use of the term “outcome metrics,” instead of “risk-based metrics” because it relates more to the costs of musculoskeletal disorders, which might be an incentive to take action [44].

Several studies report the importance of legislation to prevent work-related ill-health [29,45,46]. In the present study, the legislation was seen as the igniter to the process. Thereafter, the start-up meeting, communication, collaboration and supportive management were facilitating factors that made the process flow smoothly. In an earlier study, ergonomists reported that a start-up meeting for planning exposure assessments is important for establishing a mutual framework for the assessment and establishing contacts with the stakeholders [47]. In the present study, it was revealed that the planning at the start-up meeting should include a role and responsibility structure, and that a process leader who has the overall responsibility for the process should be appointed. A company manager should hold this role, since they have the legal responsibility for the work environment [2].

The collaboration with the ergonomist was highly appreciated. Yazdani and Wells (2018) describe that collaboration between workers and experts is needed to succeed with work environment improvements aimed at the prevention of musculoskeletal disorders [48]. The company representatives in the present study highlighted the expert competence and emphasized that the ergonomist should have both in-depth knowledge about the context of different work sectors as well as expertise in exposure assessment and the interconnection with workers’ health. Furthermore, the results reveal that, in order for the HIW-model to be feasible, the company representatives require a facilitator that guides the process leader through the different steps of the HIW-model. This further supports the description of the ergonomist’s role, as someone who initiates and guides a process, as presented by Haines et al. 2012 [49]. Hence, the ergonomist must be prepared to shoulder the responsibility as a facilitator in the process and as well as being the expert executor of the exposure assessment and the medical health checks. It is important that ergonomists (or other OHS professionals) have special knowledge and skills, as it facilitate collaboration [50] and is an incentive for employers to engage in further work place interventions [29].

The importance of communication is a well-known facilitating factor [48,50,51,52,53]. In the present study it emerged that in order to achieve an efficient flow through the process, it was crucial that the communication between the ergonomist and a designated contact person from the company went smoothly. Motamedzade et al. (2003) described that good communication between all levels within an organization is a prerequisite for enabling ergonomics improvements to be made [53]. In this study, some informants implied that the ergonomists and the company representatives did not meet their expectations regarding the cooperation and how the feedback was communicated from the ergonomist. It is important that communication between the parties be maintained even if there, because of delays or other reasons, is no progress to report [54,55]. The informants emphasized the need for straightforward reporting of the results, focusing on, based on the results from the exposure assessment and medical health checks, what actions were needed. To meet the expectations regarding communication, it might be valuable for ergonomists to pay attention to the approaches and language that are used in management system frameworks within companies. Such an approach can facilitate both communication and incorporation of musculoskeletal disorders prevention into the management system [44].

The findings highlight that the HIW-model needs to be accompanied by a process guideline, in which the aim and result of the start-up meeting are explained and that checkpoints for feedback between the executor and the company´s process leader are planned already from the start.

The tested HIW-model was the same for both the company representatives and for the ergonomist that executed the exposure assessment and medical health checks (Figure 1); hence, one should consider whether separate guidelines are needed for these different parties. The ergonomists’ experiences of the HIW-model will be explored and presented in a separate paper.

### Strengths and Limitations

In the present study a qualitative research methodology with an exploratory design, using interviews as a data collection method, was applied. Given the aims of the study, the authors believe that this approach is appropriate for gaining knowledge regarding the usability of the model, in terms of feasibility and values and for identifying impeding and facilitating factors [36]. A strength, which increases the transferability of the study [41,56], was the heterogeneity between the companies. Some were small companies with limited in-house work environment expertise and some were units of large company groups with specialized internal occupational health and safety departments. Despite the differences in size and organization, the companies had, in large, similar experiences of the HIW-model and process. This is in line with research showing that company size is not associated with perceptions of work environment prioritizations [57]. A limitation is that the included companies probably have a special interest in both work environmental issues and employee health, which causes a selection bias. However, we believe the representatives’ experiences from the study were nuanced and the results are supportive regarding the identification of deficiencies in the HIW-model and how the model can be further developed to assist the implementation of occupational health surveillance for workers exposed to hand-intensive work.

Our study lacks representatives from the public sector, which would have been valuable. In the recruitment process, we tried consciously to recruit units from the public sector, but several answered that they lacked the time to participate. Another explanation can be how the Swedish public sector utilizes OHS; findings reveal that OHS providers are seldom involved in preventive, workplace assignments [58]. The lack of representatives from a specific sector is also a finding that must be considered in the further development of the HIW-model and implementation support.

Several strategies were used to ensure trustworthiness in this qualitative study. For example, the data collection was triangulated by using both individual and focus group interviews. Furthermore, several researchers with different backgrounds were involved in the data analysis [41,56].

The present study focused on the execution of the process model, and hence the study lacks information concerning the efficacy of the model in terms of improvements in the work environments, reduced prevalence of musculoskeletal disorders in the neck and upper extremities. This is something that needs to be further studied before considering a broader implementation of the process model.

## 5. Conclusions

The company representatives appreciated the guiding HIW-model and emphasized the values of cooperating with the ergonomics expert. The model increased the risk awareness and hazardous exposure effects on musculoskeletal disorders. The holistic picture given from the interconnection between the results of the exposure assessment and the medical health checks gave an incentive for the planning of further actions. The findings reveal that a guiding process model is valuable for the understanding of occupational health surveillance and might facilitate the implementation of work environmental regulations. However, implementation support cannot only include guidance concerning the content of the main components in the process model. An important finding is that the entire execution process should be described and contain, for example, information about how a start-up meeting serves the purpose of planning the execution, and the importance to discuss and determine roles and communication paths. Based on the findings, the HIW-model needs to be developed further to serve as useful implementation support for occupational health surveillance of hand-intensive work. However, the experiences of the ergonomists also need to be explored as they, being the experts on exposure assessments and clinical examinations of the musculoskeletal system, should support the employer throughout the process.

Furthermore, the findings in this study provide knowledge regarding the content of the model components as well as the work process of occupational health surveillance, which could be applied in occupational health surveillance targeting other exposures.

## Figures and Tables

**Table 1:** Description of included companies. All company representatives except those described as “central” worked at the local site, where the occupational health surveillance took place.

No.	Organization Size (*n* of Employees)	Intervention Work Group Size (m/f)	Project Group			
			Company Representatives		Ergonomist	
			Title	Work Experience in Current Role (Years)	OHS ^a^-Provider	Work Experience in Ergonomics (Years)
1	Large > 250	12 (6/6)	First line manager	1	In-house	27
			HSE manager	2		
			Safety representative ^b^	2		
2	Medium > 50	11 (0/11)	Central HSE manager	3	External	5
			Local manager	20		
			Safety representative	28		
3	Large > 250	7 (7/0)	First line manager	20	External	9
			First-line manager	20		
			Manager	7		
4	Large > 250	26 (10/12)	First line manager	1	External	24
			First-line manager ^b^	12		
			First-line manager	-		
			HSE manager	-		
			HSE manager	-		
			Safety representatives	-		
			Safety representatives	-		
			Safety representatives	-		
5	Small < 50	11 (0/11)	Production manager	3	External	6
			First-line manager	9		
			First-line manager	2		
			Technology manager ^b^	4		
			Safety representative	-		
			Safety representative	-		
6	Medium > 50	27 (11/16)	Manager	12	External	20
			First-line manager	-		
7	Medium > 50	22 (12/10)	Production manager	-	External	14
			Safety representative	-		
8	Medium > 50	7 (4/3)	Manager	-	External	12
			HR-manager	24		
			Safety representative	34		
9	Small < 50	11 (0/11)	CEO/founder	4	External	34
			HSE manager	2		
			Central safety representative	5		
10	Large > 250	21 (0/21)	HR-manager	3	External	14
			Production manager	-		
			First-line manager	8		

^a^ OHS—Occupational health service. ^b^ Representative did not participate in any interview.

**Table 2:** Categories and sub-categories responding to the questions about experiences of the model components and facilitating factors for the execution.

**Model Components**
Identification of hand intensive work
Exposure assessment of work
The expert validity
Increased risk awareness and workplace learning
Screening and clinical examination
Feedback
Structure and presentation of feedback
Valuable for dissemination
**Facilitating factors and barriers for the execution of the model**
Planning and preparation
Communication and roles
Outer and inner contextual factors
Collaboration

## Data Availability

Data available on request due to ethical restrictions. The data presented in this study are available on request from the corresponding author. The data are not publicly available since the informants in the study have been guarantied anonymity.
